# Nerve excitability test and lead toxicity: a case-control study

**DOI:** 10.1186/s12995-023-00385-3

**Published:** 2023-09-01

**Authors:** Chung-Yao Chien, Jung-Der Wang, Chou-Ching Lin

**Affiliations:** 1 Department of Neurology, College of Medicine, National Cheng Kung University Hospital, National Cheng Kung University, 138 Sheng Li Road, Tainan, 704 Taiwan; 2 Department of Public Health, College of Medicine, National Cheng Kung University, Tainan, Taiwan; 3 Department of Occupational and Environmental Medicine, National Cheng Kung University Hospital, Tainan, Taiwan; 4 Department of Biomedical Engineering, National Cheng Kung University, Tainan, Taiwan

**Keywords:** Lead intoxication, Central conduction time, Transcranial magnetic stimulation and motor conduction, Nerve excitability test, Inward rectifying channel

## Abstract

**Background:**

Although conventional electrophysiological parameters have been proposed as clinical indicators for monitoring lead neuropathies, their correlations with blood lead level are weak. In this study, we investigated the applicability of nerve excitability tests (NETs) to evaluate lead intoxication.

**Methods:**

Fourteen workers who were exposed to lead with an elevated blood level ranging from 17.8 to 64.9 µg/dL and 20 healthy controls with similar ages and body heights were enrolled. Both workers and controls underwent nerve conduction studies (NCSs), motor evoked potentials (MEPs) with transcranial magnetic stimulation (TMS), and NETs.

**Results:**

NCSs showed prolonged distal latencies and decreased motor nerve conduction velocity of median nerves in the workers but without significant correlation to blood lead level (BLL). Significantly prolonged MEP latency was observed in the workers (+ 6 ms). NETs demonstrated hyperpolarized resting membrane potentials in stimulus-response curves and changes in the property of potassium channels under a hyperpolarized current in threshold electrotonus, implying that lead hyperpolarized nerves by interfering with potassium channels. NETs also showed a better correlation with BLL than conventional electrophysiological parameters.

**Conclusions:**

Axonal hyperpolarization and central conduction delay are more apparently reflecting elevated BLL than NCS. NET may have the potential for early detection of lead neuropathy.

## Background

Lead exposure is an occupational hazard, and lead toxicity causes multiple system dysfunction, and especially neurological manifestations [1, 2]. Abnormalities in both central [3–8] and peripheral nervous systems have been observed in traditional electrophysiological studies [1, 9–12].

In conventional electrophysiological tests of the peripheral nervous system, the results of nerve conduction studies (NCSs) including conduction velocity and amplitude of action potentials (both sensory and motor) have varied among studies, and distal latency (DL) prolongation has provided the most consistent findings [1, 7, 9–15]. Numerous studies have reported prolonged DL compared to controls, however the absolute value might be within normal limits [1, 7, 9–15]. Slowing of peripheral conduction can affect the evaluation of central conduction velocity or latency, especially when using somatosensory evoked potential studies, which represent the latency of sensory conduction from the peripheral to the central nervous system.

Electrophysiological studies have also been used to monitor or predict the clinical course in workers with subclinical exposure or low BLL [9, 10, 12, 16, 17]. However, the reported correlations between traditional parameters (NCS, DL and amplitude of compound motor action potentials) and blood lead level (BLL) have been inconsistent [1–3, 11, 15]. This is partly because the mechanisms of lead neuropathies are uncertain, although some studies have proposed interference of porphyrin metabolism [2, 8, 14] or dysfunction of ion-channels that disturb neuronal activities [18–20]. A previous study showed that the neuropathy caused by lead was predominantly the axonal type [17], however some studies have also reported segmental demyelinating characteristics [2, 21]. Pathologic findings of axonal or demyelinating processes in nerve biopsies have also been inconsistent with the results of electrophysiological parameters [21]. Therefore, alternative methods to evaluate peripheral nerve properties, such as nerve excitability tests (NETs), have been proposed. However, there has been only one case report in the literature about the application of NETs to evaluate lead toxicity [22].

Investigating the applicability of nerve excitability is warranted, because changes in the properties of ion channels at the microscopic level can precede macroscopic axonal or demyelinating pathologic processes in peripheral nerves. Thus, in this study we investigated peripheral nerve excitability in addition to conventional NCSs and motor evoked potentials (MEPs) in subjects with lead intoxication and in healthy controls. We also discussed the possibility that channelopathy is the underlying mechanism of lead neuropathy.

## Methods

### Subjects

We enrolled subjects from an occupational outpatient department who worked in a battery factory and had an elevated BLL in an annual health examination. Blood samples were tested by laboratory routine in hospital through graphite furnace atomic absorption spectrophotometry. A total of 14 workers volunteered to participate in this study, with a mean age of 50 years (range: 35 to 61 years). The average duration of lead exposure was 16 years (3–29 years) and the BLL ranged from 17.8 to 64.9 ug/dL (mean 36.76 ug/dL). Four workers complained about mild numbness over their hands, and another two complained about minor weakness of their hands. None of them had a history of diabetes mellitus or alcoholism. A control group (n = 20) of age-matched (mean age 51) subjects of white-collar workers, was recruited from hospital volunteers (served in the hospital counters and they were mostly retired teachers and business managers). The difference in body height (BH), body mass index (BMI), history of hypertension, impaired fasting glucose (IFG) state and alcohol consumption between the workers and controls was insignificant. Smoking habit was more frequent in workers (Table 1). All participants were right-handed without known neurological disorder.

**Table 1:** Basic characteristics of the workers and controls

	Workers (n = 14)	Controls (n = 20)	p value (Fisher)
Sex (male/female)	12/2	8/12	0.01
Age (year range)	51 (35–61)	56 (27–69)	0.13
Body height (cm)	162.9 ± 6.3	161.7 ± 7.4	0.64
BMI	23.09 ± 3.33	23.09 ± 2.37	0.99
Hypertension	5	2	0.10
IFG	1	1	1.00
Alcohol consumption	3	4	1.00
Smoking	8	0	< 0.01
Blood lead level	36.76 ± 12.33	2.4 ± 0.74	0.00

BMI: body mass index; IFG: impaired fasting glucose;

Ethics approval for the study was obtained from the National Cheng Kung University Hospital Institutional Review Board research ethics committees. All workers and controls were informed of the objective and the procedure of this study, and all signed a written consent form.

### Evaluation methods

#### Nerve conduction study

The stimulating electrode was placed on surface of targeted site and square-wave pulses ranging from 0.1 ~ 0.5 ms and 10 to 50 mA was given. At each site, 5 to 10 stimuli of trial ramping from 0.1 ms and 10 mA until the maximal response of compound muscle action potential (CMAP) was obtained. Motor nerve conduction velocity (MNCV) and distal motor latency of the median nerve were determined in the dominant upper limb. The median nerve was stimulated at the wrist (3 cm proximal to the distal crease of the wrist) and at the elbow. The CMAP of the thenar muscles was recorded with surface disc electrodes, and DL was defined as the measured time interval between the artifact caused by stimulation at the wrist and the onset of action potential at the thenar muscles. Besides distal conduction of peripheral nerves, proximal conductions (F-wave with M-wave) were also measured. The conduction latency from spinal cord to the stimulation site was estimated with the formula (F-M-1)/2. The stimulation and recording of nerve conduction test was performed by the equipment of Natus Viking™ On Nicolet® EDX.

#### Nerve excitability test

We followed the TROND protocol [23] for the threshold-tracking technique [24] using QTRAC software (Institute of Neurology, Queen Square, London, UK) [25]. CMAPs were recorded using surface electrodes at the thenar muscles by stimulation at the wrist, as with the NCSs. The automated excitability evaluation protocols included the following four measures.

Stimulus-response (SR) curve showed the response as the stimulus was increased from zero to where it produced the maximal potential. The maximal value was used in later tests, and 50% of maximal response was used to normalize other responses in the SR curve. Fifty stimuli were given in SR curve protocol. The current–threshold relationship (I/V relationship) was obtained as follows. The threshold was identified using 1-ms test stimuli applied 200 ms after the onset of a long-lasting subthreshold polarizing current, the strength of which was altered in 10% steps from + 50% (depolarizing) to − 100% (hyperpolarizing) of the control threshold. This current–voltage relationship depended on the rectifying properties of the nodal and internodal axolemma. Including conditioning stimuli, in total 34 stimuli were applied. Threshold electrotonus (TE) reflected the underlying changes in membrane potential. Prolonged subthreshold currents were used to alter the potential difference across the internodal axonal membrane. The subthreshold polarizing currents were of 100-ms duration and set to be + 40% (depolarizing, TEd) and − 40% (hyperpolarizing, TEh) of the control threshold current. Threshold was tested at different time points during and after the 100-ms polarizing currents. In the hyperpolarizing protocol, when the current had been applied for 20–40 ms (30–50 ms on the time scale), the tempered slope (S1) reflected activities of potassium channels; and from 90 to 100 ms (100–110 ms on the time scale), the flattened curve (S3) reflected activities of inward rectifying channels Including conditioning stimuli, in total 208 stimuli were given. Recovery cycle (RC) measured the recovery of axonal excitability following the delivery of a supramaximal conditioning stimulus with conditioning-test intervals from 2 to 200 ms. The refractory period at short conditioning-test intervals was due to the inactivation of transient Na^+^ channels with a resultant increase in threshold. This was followed by a period of superexcitability due to the depolarizing afterpotential. The late-subexcitable period during which axonal excitability was reduced reflected the kinetics of voltage-dependent slow K^+^ channels activated by the conditioning stimulus. Including conditioning stimuli, in total 36 stimuli were applied. The stimulator (DS5 isolated bipolar stimulator developed by Digitimer Ltd), data acquisition (National Instruments (NI) with BNC terminals) and amplifier (The D440 amplifier designed by Digitimer) were identical to the instruments mentioned in the consensus guideline [25].

#### TMS/central conduction time

MEPs were determined in response to transcranial magnetic stimulation (TMS) applied to the contralateral primary motor cortex. Monophasic single-pulse TMS (Magstim 200² system, Magstim Company Ltd., Spring Gardens, Whitland, UK) was applied to the scalp position around the vertex through a circular coil to evoke adequate amplitude of CMAPs, which were recorded over the right forearm, at the bulk of extensor digitorum superficialis of the right forearm, around 15 cm proximal to the lateral styloid process. Motor threshold of MEP was determined TMS placed tangentially at the vertex with the handle pointed backwards and laterally at an angle of 45° from the midline. The initial magnitude of magnetic stimulation was set to 30% of the maximal output power, and then increased in increments of 5% until a response was visually identified. Then, the magnitude of stimulation was adjusted in increments of 1% with the goal of finding the maximal magnitude at which the response was valid fewer than 5 times. The smallest magnitude that fulfilled the above criteria was defined as the motor threshold. MEP latency was calculated as the time interval from the start of stimulus by TMS to the onset of CMAPs.

### Statistical analysis

Shapiro-Wilk test indicated that the collected data did not violate the assumption of normal distribution (p > 0.05). Comparisons of all electrophysiological parameters between the workers and controls were performed using the unpaired t-test. Pearson’s correlation coefficient r represented the level of correlation with BLL. Linear regression analysis and multivariate regression model were then applied. All statistical analyses were performed using SPSS software (SPSS Statistics for Windows, Version 17.0, SPSS Inc., Chicago, USA).

## Results

The age, BH, motor and sensory symptoms, DL of median nerve conduction velocity studies, MEP latency, and parameters with a significant difference (p < 0.05) in NETs of the workers are shown in Table 2. Detailed analysis is discussed separately below.

**Table 2:** Characteristics, clinical manifestations, blood lead levels and selected test results of the workers

			Symptom		Lead content			Median MNCV		Evoked potential	NET		
No	Age (yr)	BH (m)	S	M	BLL (µg/dL)	Exp. (yr)	Cumulative lead (µg/dL*yr)	DL (ms)	MNCV (m/s)	MEP latency (ms)	Stim. 50% max resp. (mA)	S1 (TEh20-40 ms) (%)	S3 (TEh90-100 ms) (%)
1	35	1.53	+	-	38.7	8	309.6	3.05	56.00	24.86	4.31	-101.09	-133.25
2	48	1.75	+	-	40.3	14	564.2	3.75	56.30	25.14	7.15	-85.22	-117.87
3	47	1.69	-	-	35.9	23	825.7	3.80	56.10	23.71	4.17	-90.67	-120.16
4	48	1.60	-	-	33.7	2.5	84.25	5.25	55.20	23.57	6.59	-103.27	-155.06
5	52	1.55	+	-	21.3	3	63.9	4.60	50.60	22.00	4.61	-83.28	-91.67
6	50	1.64	-	-	43.3	22	952.6	3.90	49.50	27.29	6.70	-95.92	-142.78
7	61	1.58	-	-	20.6	5	103	3.40	55.30	22.29	5.37	-101.69	-130.87
8	55	1.62	-	+	33.5	23	770.5	3.05	57.60	22.29	3.59	-96.02	-131.15
9	54	1.69	+	+	17.8	21	373.8	4.10	57.10	22.86	4.88	-78.09	-103.74
10	54	1.58	-	-	64.9	18	1168.2	3.10	57.10	23.00	4.20	-99.53	-148.18
11	43	1.65	-	-	31.5	21	661.5	3.45	60.80	20.14	3.81	-101.90	-158.68
12	54	1.59	-	-	39.1	29	1133.9	2.70	56.00	22.57	4.51	-103.88	-147.34
13	54	1.63	-	-	47.8	29	1386.2	3.85	54.30	26.29	5.18	-106.53	-138.62
14	45	1.70	-	-	46.3	8	370.4	3.65	57.30	22.57	4.32	-100.95	-136.96

NET: nerve excitability test; BH: body height; BLL: blood lead level; Exp: lead exposure history. DL: distal latency; MNCV: motor nerve conduction velocity; MEP: motor evoked potential; S: sensory symptoms; M: motor symptoms; Stim. 50% max resp.: stimulus for 50% of max response; yr: year

### Nerve conduction study

There was a relative increase in DL in the workers’ median nerves compared to the controls (p = 0.002) and a decrease in MNCV (p = 0.037). The absolute values of MNCV in both groups were within the normal range. Although three workers had prolonged DL that mildly exceeded the normal limit, the mean DL of the workers was within the normal range (< 4 ms). In the F-wave study, latencies from spinal cord to the stimulation site (F-M-1)/2 were 10.44 ± 0.53 ms and 10.76 ± 1.15 ms in controls and workers, respectively. The difference between groups was not significant (p = 0.446) (Table 3).

**Table 3:** Correlations between blood lead level and electrophysiological parameters, motor evoked potentials and indices of nerve excitability test

	Parameters	Controls (± SD)	Workers (± SD)	p value
NCS	Distal latency (ms)	3.06 (± 0.27)	3.69 (± 0.64)	0.002
	Distal CMAP amplitude (mV)	7.55 (± 0.65)	6.63 (± 1.97)	0.108
	MNCV (m/s)	58.14 (± 6.35)	55.66 (± 2.72)	0.037
	Proximal latency (ms)^#1^	10.44 (± 0.53)	10.76 (± 1.15)	0.446
MEP	Latency (ms)	17.44 (± 9.73)	23.47 (± 1.80)	0.000
NET	Stimulus-Response curve			
	Stim. 50% max resp. (mA)	3.66 (± 2.52)	4.96 (± 1.08)	0.001
	SR slope	7.45 (± 5.47)	5.13 (± 1.61)	0.003
	Peak response (mV)	2.03 (± 1.05)	2.93 (± 0.91)	0.014
	Threshold electrotonus			
	S1 (TEh 20–40 ms)	-90.63 (± 48.03)	-96.29 (± 8.38)	0.046
	S3 (TEh 90–100 ms)	-119.22 (± 64.85)	-132.59 (± 18.30)	0.027

^#1^ The conduction time from spinal cord to the stimulation site

SD: standard deviation; BLL: blood lead level; NCS: nerve conduction study; MEP: motor evoked potential; NET: nerve excitability test; CMAP: compound muscle action potential; MNCV: motor nerve conduction velocity; SR slope: stimulus-response slope; TEh: hyperpolarization condition of threshold electrotonus; r: correlation coefficient; Stim. 50% max resp.: stimulus for 50% of max response

### Motor evoked potential

The mean MEP latency in the workers (23.47 ± 1.87 ms) was longer than that in the controls (17.44 ± 2.47 ms), and the difference was statistically significant (p < 0.001) (Table 3). It should be mentioned that the BHs of the two groups were similar (workers: 162.86 ± 6.26 cm, controls: 161.72 ± 7.84 cm, p = 0.69). In addition, the recording site of the MEPs was located over the bulk of the extensor digitorum superficialis, around 15 cm proximal to the lateral styloid process of the forearm and close to the elbow. This was even more proximal to where the DL of the radial nerve is usually measured (8–10 cm proximal to the styloid process). Thus, a prolonged DL in the hand segment would have a very limited contribution to this result. Besides, correlation analysis between MEP and latency from spinal cord to the stimulation site showed insignificant correlation (r = 0.419, p = 0.136). This may imply that the difference of MEP latency between groups represent the difference of central conduction.

### Nerve excitability test

**Fig. 1:**
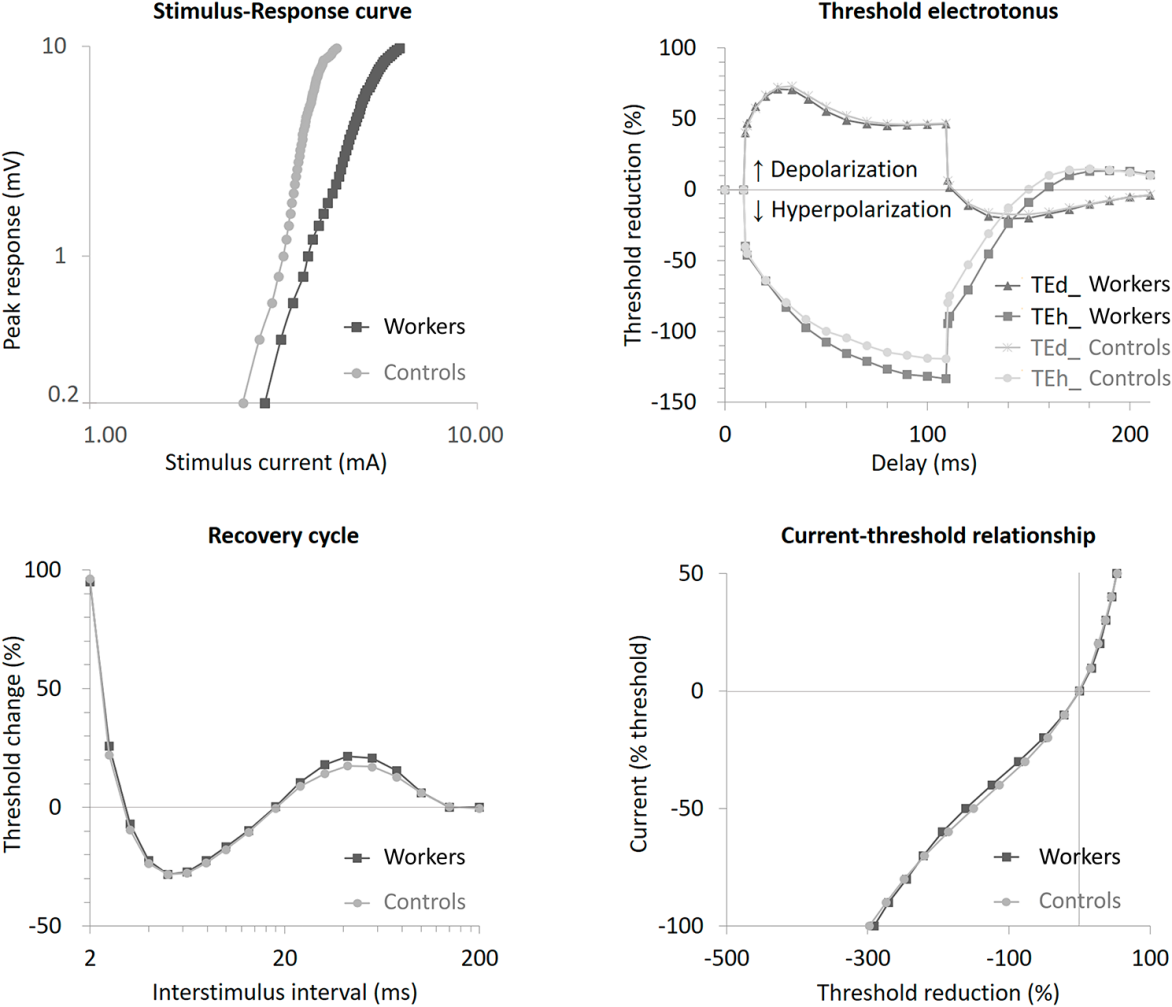
Mean group results of NETs including SR curve, TE, RC and I/V slope. Dark square: workers; light circle: controls. Left upper: hyperpolarization of the resting membrane potentials was found in SR curve. Right upper: The curve in hyperpolarized conditions for the worker group was significantly deviated downward starting from the S1 phase and most separated in the S3 phase, then gradually became closer to the curve of the control group during the overshoot phase. Left lower: The curve during the subexcitability phase was slightly deviated upward in the worker group. Right lower: Although only segmental (-20~-40%) left deviation during hyperpolarized, the difference was significant (p = 0.03). The overall interpretation suggests that the resting membrane potential was hyperpolarized because of the tendency of over-expressed potassium channels with relatively adequate inward rectifying channels

NETs demonstrated changes in hyperpolarization in SR (stimulus-response) curve and TE (threshold electrotonus) in the workers (Fig. 1). Indices with significant differences between the workers and controls included: (1) stimulus for 50% max response (workers: 4.96 ± 1.08, controls: 3.66 ± 2.52, p = 0.001), (2) slope of stimulus-response (workers: 5.13 ± 1.61, controls: 7.45 ± 5.47, p = 0.003), and (3) peak response (mV) (workers: 2.93 ± 0.91, controls: 2.03 ± 1.05, p = 0.014) in the SR curve. This reflected the resting membrane potentials, which in turn reflected the summed actions of various types of sodium and potassium channels. Other significant differences were observed, especially the hyperpolarization segment of threshold eletrotonus, TEh), in (1) S1 (TEh 20–40 ms) (workers: -96.29 ± 8.38, controls: -90.63 ± 48.03, p = 0.046) and (2) S3 (TEh 90–100 ms) (workers: -132.59 ± 18.30, controls: -119.22 ± 64.85, p = 0.0270), which were due to potassium channels and inward rectifying channels (Table 3). In general, there was no significant depolarization or hyperpolarization in the I/V (current-threshold) relationship and RC (recovery cycle). However, small changes in the I/V relationship manifested as a slight left deviation in the hyperpolarized portion of the curve under − 20 to -30% of the threshold current (workers: -86.78 ± 17.47, controls: -77.29 ± 11.15, p = 0.03). A small increase in subexcitability (workers: 17.92 ± 11.94, controls: 14.24 ± 5.77, p = 0.12) was also observed in the RC with no decrease in superexcitability.

**Fig. 2:**
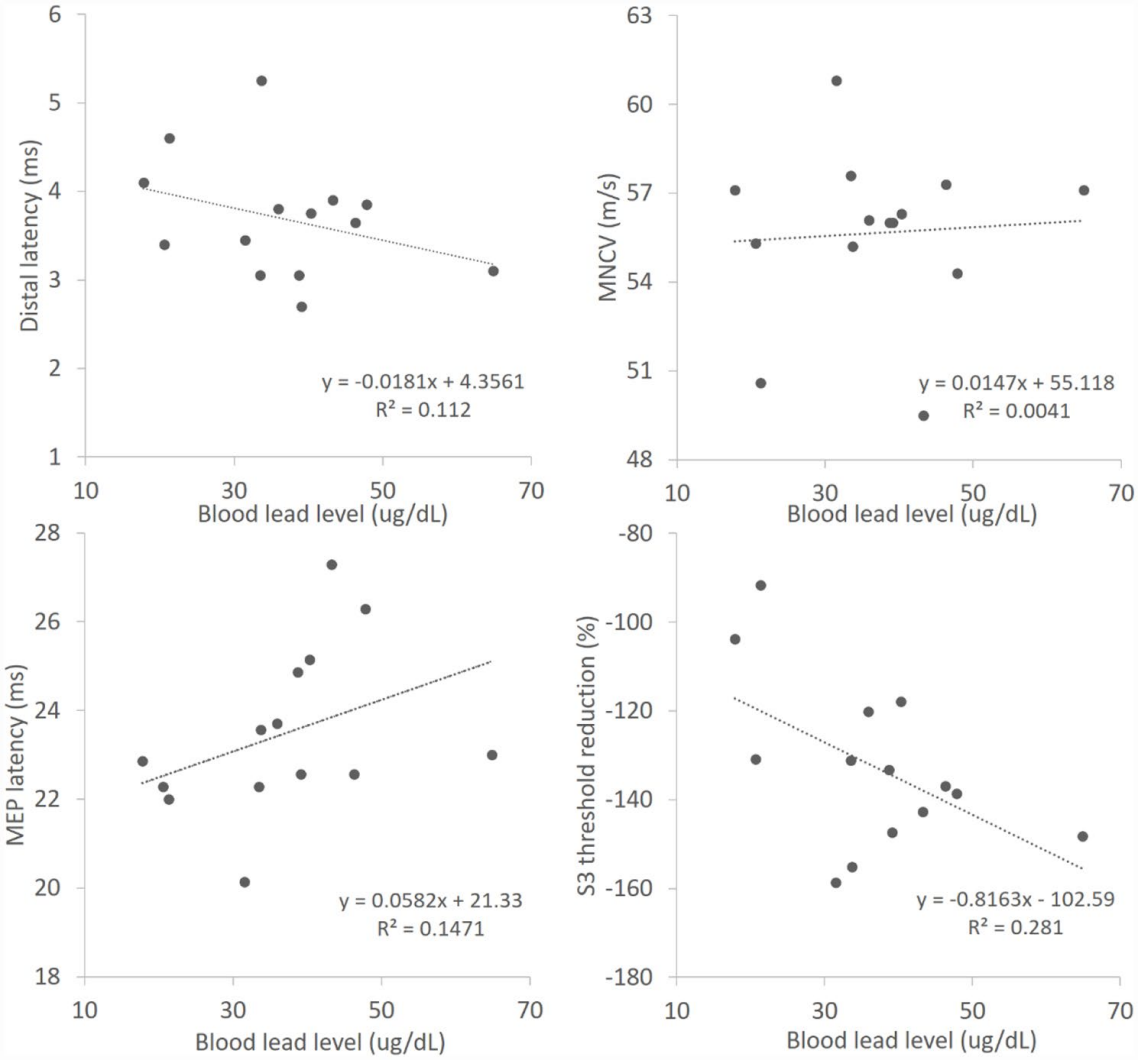
Linear regression of the results of the NCSs, MEPs and indices of NETs to the BLL. S3 (TEh 90–100 ms) was better correlated with the BLL than the other three parameters. DL: distal latency; BLL: blood lead level; MNCV: motor nerve conduction velocity; MEP: motor evoked potential; TEh: hyperpolarization condition of threshold electrotonus

### Correlation to blood lead level

The correlations between BLL and electrophysiological parameters were calculated (Fig. 2). S3 (TEh 90–100 ms) of TE in NET (r = -0.53) was most linearly correlated with BLL. S1 (TEh 20–40 ms) also had a moderate correlation with BLL (r=-0.46). These two parameters represented functional changes in slow K channels and inward rectifying channels. Parameters in the SR curve showed weak or no correlations (stimulus for 50% max response, r = -0.01; stimulus-response slope, r = 0.00; peak response (mV), r = 0.37). Relatively weak correlations to BLL were demonstrated in DL (r = -0.33), MNCV (r = 0.06) and MEP latency (r = 0.38). Linear regression analysis of selected parameters (with r > 0.3 including DL, MEP, peak response, S1 and S3) on BLL was obtained. Only S3 (TEh 90–100 ms) which representing inward rectifying channel function showed significance (p = 0.046). In one study on nerve excitability test [26], it reported that the parameters we focused on did not differ between genders. In the model adjusted for smoking, inward rectifying channel (S3) turned out to be less significantly correlated to BLL (Table 4). As seen in Table 2, the cumulative BLL index (µg/dL*yr) did not correspond to all electrophysiological studies.

**Table 4:** Regression Analysis of parameters on blood lead level in workers, unadjusted and adjusted smoking

		Univariate analysis			Multivariate analysis		
		p value	R^2^ (adjusted R^2^)	Standardized *β* (95% CI)	p value	R^2^ (adjusted R^2^)	Standardized *β* (95% CI)
NCS	Distal latency (ms)	0.242	0.112 (0.038)	-0.335 (-0.927, 0.258)	0.216	0.225 (-0.120)	-0.431 (-1.163, 0.302)
MEP	Latency (ms)	0.176	0.147 (0.076)	0.384 (-0.197, 0.964)	0.305	0.195 (-0.163)	0.359 (-0.387, 1.106)
	Peak response (mV)	0.187	0.140 (0.068)	0.374 (-0.209, 0.958)	0.321	0.370 (0.091)	0.307 (-0.353, 0.967)
NET	S1 (TEh 20–40 ms)	0.094	0.216 (0.151)	0.465 (-1.022, 0.092)	0.252	0.247 (-0.088)	-0.390 (-1.112, 0.331)
	S3 (TEh 90–100 ms)	0.051	0.281 (0.221)	-0.530 (-1.063, 0.003)	0.237	0.482 (0.252)	-0.335 (-0.934, 0.263)

The multivariate model adjusted age, gender and smoking

### Correlation to clinical symptoms

Only two workers (cases 5 and 9) with a prolonged DL had mild subjective hand numbness, one (case 9) of whom also had minor subjective weakness. Neither of the other two (cases 1 and 2) workers with hand numbness nor the other one (case 8) worker with minor hand weakness had a prolonged DL. In the NET part, none of parameters correlated to clinical symptoms. The BLL or even cumulative BLL index (µg/dL*yr) did not correlate to clinical symptoms.

## Discussion

The increased DL and decreased nerve conduction velocity in the workers with lead intoxication observed in this study have been well-established in past studies. However, in this study the relationship between DL or nerve conduction velocity between BLL was not apparently demonstrated. The delayed response in evoked potentials in prior studies is also consistent with the prolonged MEP latency in our workers. The new findings in this study are the changes in properties of the potassium channels over the motor component of the median nerve as reflected by S1 and S3 of TE in NET, which correlated well with BLL. In addition, the correlation strength according to Pearson’s correlation coefficient (r = -0.53) was better than the correlations of parameters in previous conventional electrophysiological studies in peripheral nerves (mostly r < 0.5) [7, 15, 16]. Linear regression analysis also supported the significant correlation. Therefore, indices of NETs may be reliable indicators to evaluate early changes before developing into lead neuropathy. After adjusting confounding factors of smoking that were unevenly distributed between groups, the significance then decreased. This probably resulted from the small sample size.

Furthermore, NET data can help to exclude the effects of carpal tunnel syndrome. Some researchers [27] have reported that the increased DL in median NCSs may result from a mechanical effect or compressive neuropathy such as carpal tunnel syndrome, which is difficult to distinguish using conventional NCSs. NETs evaluate changes in membrane potentials and activities of ion channels, and provide additional information to differentiate the nature of neuropathy. That is, carpal tunnel syndrome presents as depolarization of membrane potentials resulting from compression, and partially from an ischemic effect due to Na^+^/K^+^ ATPase dysfunction [28–31]. In the current study, the overall presentation of NETs indicated hyperpolarization in the SR curve and dysfunction of various potassium channels as reflected in TE. In a clinical setting, this may provide an additional approach to distinguish carpal tunnel syndrome from lead and other toxic neuropathies [7, 32] if conventional electrophysiological tests only show prominent DL prolongation. The prolonged DL in the median nerves of our workers was probably merely due to a compression effect which depolarized membrane potentials.

Several studies have reported central nervous system involvement in lead toxicity through electroencephalography, somatosensory evoked potentials, brainstem auditory evoked potentials, visual evoked potentials and P300, which suggests possible cortical or subcortical dysfunction. To identify functional changes in motor system related to lead toxicity, in addition to peripheral electrophysiological tests, MEPs provide clearer and more independent observations [33]. Before NCS parameters of peripheral nerves become abnormal or prolonged latency from spinal cord to the stimulation site, motor propagation in the central nervous system has already significantly slowed. Our data did not reveal a strong correlation between prolonged MEP latencies and symptoms of weakness as reported in previous reports [14]. Therefore, the clinical symptoms or signs of weakness in lead intoxication could be caused by dysfunction of central motor pathways with normal or obscure changes in peripheral NCSs.

Before our investigation, only one case report has used NET to study lead intoxication, which showed an increase in threshold (hyperpolarization) in the SR curve and TE in acute lead intoxication [22]. We also found similar findings in our 14 workers. Hyperpolarized resting membrane potentials have been reported in amyotrophic lateral sclerosis, Charcot-Marie-Tooth disease 1 A, multifocal motor neuropathy with conduction block, hypokalemia, and acute intermittent porphyria [34, 35]. Similar hyperpolarization of downward S3 phase has been reported in hypokalemia and potassium channelopathy (KV1.1), however, other variables did not shift as the finding in this study. The one common toxic substance causing hyperpolarization of downward S3 phase is oxaliplatin which also acts in a complicated way to lead to neuropathy. Therefore, lead toxicity appears to involve more complex interactions with potassium and inward rectifying channels, resulting in neuropathies. This point has seldom been mentioned in previous reports, because the generally accepted theory about lead toxicity is the competition of potassium ions with calcium ions through various mechanisms [36].

Unexpectedly, the hypothesis of disturbing calcium ion metabolism could not completely explain our MEP or NET findings. MEP latency relies on the corticospinal tract itself but not on the function of releasing neurotransmitters in synapses [36]. Calcium ion channel-related inward rectifiers (G-protein coupled and ATP-sensitive) are mainly distributed in the central nervous system and heart/vagus nerve. In the peripheral nerves, inward rectifiers are hyperpolarized by activated cyclic nucleotide-modulated (HCN) channels [37] functioning with minimal influence of calcium metabolism. Furthermore, besides channelopathy per se, a resultant decrease in S3 accommodation in TE secondary to the central motor neuropathy [38] may also play a role in changes in inward rectifiers. Our findings imply a scenario of more complicated pathophysiology in lead neurotoxicity other than solely by the metabolism of calcium ions.

Finally, compared with conventional NCSs, NETs demonstrated better sensitivity in detecting lead intoxication, and a better correlation with BLL. However, the significance of many electrophysiological results reduced after adjusting gender, age and smoking (Table 4). The small sample size is a crucial limitation that the significance of correlation between BLL and hyperpolarization of NET decreased after adjusting confounders, such as uneven distribution of smoking between groups. So far, effects of smoking on nerve excitability has not been studied yet [25]. To standardized various electrophysiological examination (such as limbs surface temperature when stimulation) and to establish the relationship between them was also a challenging issue (such as from central to peripheral, sensory and motor nerve studies correlation to clinical symptoms). Further investigations are warranted to investigate whether potassium channelopathy or reciprocal changes in central and peripheral neuropathies are the mechanism underlying lead neurotoxicity.

## Conclusions

Axonal hyperpolarization and central conduction delay are more apparently reflecting elevated BLL. Central conduction delay exists in subjects with increased lead absorption. We propose that NETs may have potentials for providing biomarkers to evaluate and follow patients with lead neuropathy. Indices reflecting interference of potassium channels were better correlated with BLL than parameters derived from conventional electrophysiological tests. This study provides a potential direction for further investigations into the pathophysiology of neuropathy caused by lead intoxication.

## Data Availability

Data available on request from the authors.
